# Addition of Graphene Oxide in Different Stages of the Synthesis of Waterborne Polyurethane-Urea Adhesives and Its Influence on Their Structure, Thermal, Viscoelastic and Adhesion Properties

**DOI:** 10.3390/ma13132899

**Published:** 2020-06-28

**Authors:** Abir Tounici, José Miguel Martín-Martínez

**Affiliations:** Adhesion and Adhesives Laboratory, Department of Inorganic Chemistry, University of Alicante, 03080 Alicante, Spain; abirbibo27@gmail.com

**Keywords:** waterborne polyurethane-urea adhesive, graphene oxide, structure-property relationship, adhesion, thermal properties, viscoelastic properties

## Abstract

In this study, 0.04 wt % graphene oxide (GO) was added in different stages (before and after prepolymer formation, and during water addition) of the synthesis of waterborne polyurethane-urea dispersions (PUDs) prepared by using the acetone method. The structural, thermal, mechanical, viscoelastic, surface and adhesion properties of the polyurethane-ureas (PUUs) containing 0.04 wt % GO were studied. The addition of GO before and after prepolymer formation produced covalent bonds between the GO sheets and the NCO groups of the isocyanate, whereas the GO sheets were trapped between the polyurethane chains when added during water addition step. As a consequence, depending on the stage of the PUD synthesis in which GO was added, the degree of micro-phase separation between the hard and soft segments changed differently. The addition of GO before prepolymer formation changed more efficiently the polyurethane-urea structure, i.e., the covalently bonded GO sheets disturbed the interactions between the hard segments causing lower percentage of free urethane groups, higher crystallinity, lower storage modulus, higher yield stress and T-peel strength. The interactions between the GO sheets and the polymeric chains have been evidenced by plate-plate rheology, thermal gravimetric analysis and spectroscopy. On the other hand, physical interactions between GO and the polyurethane-urea chains were produced when GO was added in water during the synthesis, i.e., GO was acting as a nanofiller, which justified the improved mechanical properties and high lap-shear strength, but poor T-peel strength.

## 1. Introduction

Polyurethanes (PUs) are widely used as coatings and adhesives, and particularly the waterborne polyurethane-urea dispersions (PUDs) are currently used in flexible coatings and adhesives due to environmental regulations concerning the organic solvent emissions. PUD typically consists of nano-sized particles dispersed in water due to the presence of ionic groups on the surface (polyurethane-urea ionomer) and they are synthesized by reacting a polyol with a diisocyanate, an internal emulsifier and a chain extender. Typically, an NCO-tipped prepolymer ionomer is first prepared, then dissolved in acetone, while a chain extender, such as a diamine, is then added to react with the terminal NCO groups and increase the molecular weight. To make the polyurethane dispersible in water, the acetone is removed to produce an aqueous polyurethane-urea dispersion [1]. The waterborne polyurethane-ureas (PUUs) show segmented structures consisting of soft and hard segments, as well as ionic interactions. The hard and soft segments are incompatible, resulting in phase separation due to the formation of hard and soft micro-domains in the PUUs, the degree of micro-phase separation determines their properties [2].

Graphene, a monolayer of two-dimensional sp^2^ bonded carbon atoms sheet, possesses excellent strength (130 GPa), high modulus (1000 GPa) [3], a large surface area (2600 m^2^/g), remarkable flexibility, high thermal stability, high thermal conductivity (4.84 × 10^3^–5.30 × 10^3^ W/mK) [4,5,6] and high electrical conductivity (around 6000 S/m) [7]. Among the different preparation methods of graphene, the chemical reduction of graphene oxide (GO) layers has been the most frequently researched route, owing to their cost-effectiveness, scalability and production in bulk quantities [8]. During recent years, graphene has been added to different polymers for improving the performance of composites [9], batteries and sensors [10], among others, because the addition of a low volume fraction of graphene improved noticeably their mechanical, electrical and thermal properties [11]. In particular, graphene nanofiller have been added to polyurethanes, and their improved properties have been ascribed to the interactions between the polyurethane chains and the surface functional groups of the graphene sheets [12,13,14].

Previous studies have revealed the advantages of adding carbon nanofillers into solvent-born thermoplastic polyurethanes; i.e., polyurethanes dissolved in organic solvents [15,16,17]. However, few studies have considered the use of graphene derivatives into waterborne polyurethanes [18,19,20,21,22,23,24] mainly due to the poor ability of the graphene to disperse in water, in which it tends to agglomerate. Three different general methods have been proposed for improving the dispersion of graphene in polyurethanes, the solution mixing [25], the melt blending [26], and the in-situ polymerization [27].

The electrical conductivity of waterborne polyurethanes improved by adding of 1 or 4 wt % GO, the mechanical and thermal properties also increased [19,20]. Lee et al. [21] have shown increased degradation temperature of waterborne polyurethane containing different amounts of functionalized graphene, the improvement was ascribed to enhanced crystallization of the soft segments and decreased interactions between the hard segments of the polyurethane. Kim and Lee [22] showed that the graphene-waterborne polyurethane films containing 2–16 wt % graphene prepared in 25 wt % distilled water improved their electrical and thermal properties. Zhang et al. [23] have added amine-modified GO to improve the water resistance, the thermal, and the mechanical properties of waterborne polyurethanes, and similarly Wan and Chen [24] dispersed GO in deionized water under sonication for 2 h and added to waterborne polyurethane by mechanical stirring for improving its thermal stability and mechanical properties.

There are few studies considering the influence of adding graphene derivatives on the properties of adhesives. Fu et al. [28] prepared graphene-epoxy adhesive by expanding the graphene layers for imparting thermal conductivity and increase adhesion. Similarly, Araldite 2011 epoxy adhesive has been reinforced by adding different amounts of reduced graphene oxide (r-GO), the addition of 0.5 wt % r-GO enhanced the tensile strength, and an increase of 27 % in single lap-shear strength was obtained [29]. On the other hand, Choi et al. [30] improved the thermal conductivity of poly(methyl methacrylate) adhesives by adding 0.09–0.9 wt % graphene. 

The addition of graphene derivatives for improving the adhesion and surface properties of waterborne polyurethane coatings has also been scarcely studied. Thus, Zhao et al. [31] have prepared waterborne polyurethane coatings containing 0.1–1 wt % polydopamine functionalized graphene by solution blending, the coatings made with 0.5 wt % functionalized graphene improved the corrosion resistance, hydrophobicity, and adhesion. Nine et al. [32] prepared graphene composite by growth of sodium metaborate crystals at the interface and between the graphene layers during GO reduction intended as fire-retardant coatings for metal and glass, upon which they found good cross-hatch adhesion and high mechanical strength; the enhanced adhesion was ascribed to strong binding between the native oxide layer on the metal surface and the borate groups. Similarly, the mechanical and thermal properties of waterborne polyurethane coatings modified with cellulose nanocrystals and graphene by using a sol method have been studied by Yang et al. [33] improved hardness, abrasion resistance, high thermal conductivity, and adhesion. Kale et al. [34] mixed a commercial waterborne polyurethane with 0.2 wt % graphene oxide and silica nanocomposites intended for leather coatings and improved mechanical and thermal properties, flexibility, and adhesion were obtained, while the enhanced adhesion was ascribed to improved interfacial interactions. 

To the best of our knowledge, the addition of graphene derivative for improving the adhesion of polyurethane adhesives has been only considered by Cristofolini et al. [35]. These authors prepared 0.01 wt % carboxyl-functionalized graphene platelets (GP) and 0.1 wt % GO blends that were added to very diluted in water (1 wt %) commercial waterborne polyurethane dispersion, after which an increase in the peel strength was obtained. In a contrasting approach, in this study, the adhesion of waterborne polyurethane-urea adhesives (PUDs) was modified by adding GO only, and in an amount lower than 0.1 wt % during their syntheses; this methodology has not been considered yet in the existing literature for improving the adhesion properties of polyurethanes. Thus, 0.04 wt % GO was added in different stages of the synthesis of the PUDs by using the acetone method; this amount of GO was optimal on the basis of previous study [36]. In-situ polymerization of the GO sheets and the polyurethane chains was produced by adding GO before and after prepolymer formation during PUD synthesis, and solvent mixing of the GO sheets and the polyurethane chains added was produced when GO was incorporated in water during PUD synthesis. Due to the interactions between the GO sheets and the polyurethane-urea chains being changed dramatically, this led to different micro-phase separation and adhesion properties. The properties of the different GO-waterborne polyurethane-urea composites were studied and their adhesion properties were assessed under peel and shear stresses.

## 2. Materials and Methods

### 2.1. Materials

Different reactants were used in the synthesis of the waterborne polyurethane-urea dispersions (PUDs). Polyadipate of 1,4-butanediol with molecular weight of 2000 Da—Hoopol F-501 (Synthesia, Barcelona, Spain) was used as polyol, and its residual moisture was removed by heating at 80 °C under 300 mbar for 2 h. Isophorone diisocyanate (IPDI), 2,2 bis(hydroxymethyl) propionic acid (DMPA) internal emulsifier, triethylamine (TEA) neutralization agent, monohydrated hydrazine (HZ) chain extender—60 wt % purity -, and dibutyltin dilaurate (DBTDL) catalyst were used; all these reactants were supplied by Sigma Aldrich (Barcelona, Spain). Acetone (Panreac, Barcelona, Spain) and deionized water were used. Graphene oxide (GO) slurry with a concentration of 30 g GO/L was supplied by Graphenea (San Sebastián, Spain); the nominal solid GO content in the slurry was 3 wt %.

### 2.2. Synthesis of the Waterborne Polyurethane-Urea Dispersions without and with 0.04 wt % GO

**Scheme 1:**
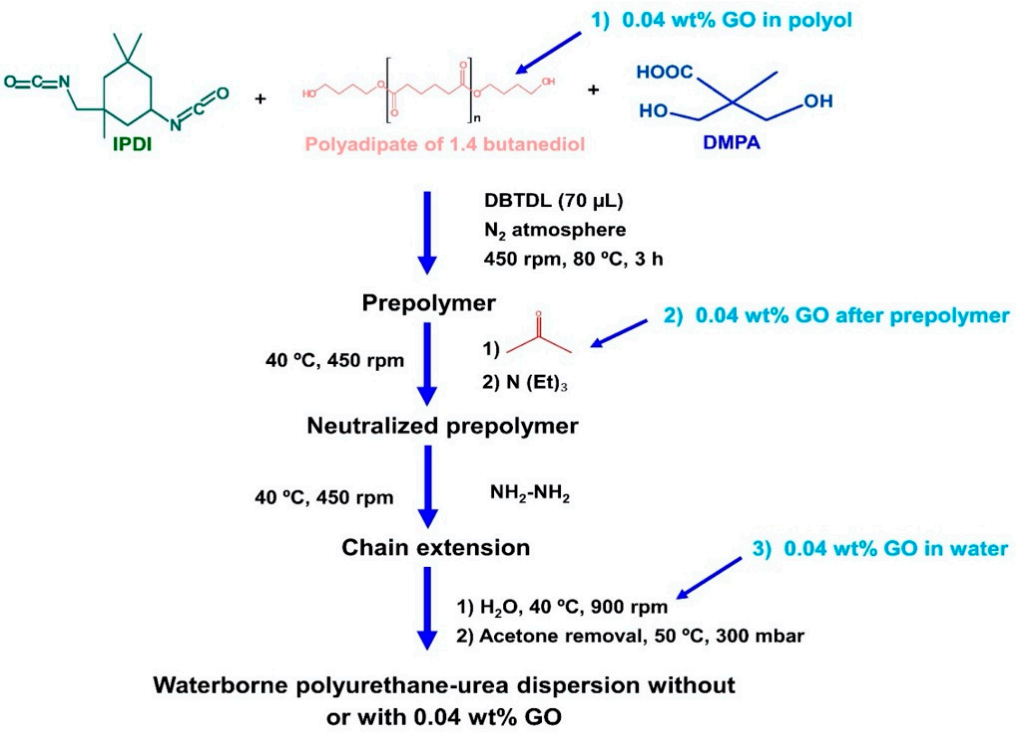
Synthesis of the waterborne polyurethane-urea dispersions without and with 0.04 wt % GO added in different stages of the synthesis.

The waterborne polyurethane-urea dispersions (PUDs) were synthesized by using the acetone method, an NCO/OH ratio of 1.5 was used, and the targeted solids content was 40 wt %. One PUD without GO was synthesized as reference and three PUDs containing 0.04 wt % GO added in different stages of the synthesis were also prepared. Scheme 1 shows a scheme of the synthesis procedure. 

The PUD without GO was synthesized by following different consecutive stages:

(a) Synthesis of the prepolymer: The polyol, 5 wt % DMPA (with respect to the total amount of prepolymer) and DBTDL catalyst were added into the reactor at 80 °C under mechanical stirring at 450 rpm for 30 min. Then, IPDI diisocyanate was added slowly, allowing the reaction for 2 h 30 min.
(b) Dissolution of the prepolymer in acetone: The temperature was lowered to 40 °C and the acetone was added to dissolve the prepolymer, maintaining the mechanical stirring at 450 rpm for 30 min.
(c) Neutralization of the ionic groups: TEA in 25 mL acetone was added to neutralize the protons of the DMPA hard segments in the prepolymer under mechanical stirring at 40 °C and 450 rpm for 30 min.
(d) Chain extension: HZ was added under mechanical stirring at 40 °C and 450 rpm for 30 min.
(e) Dispersion in water: The stirring speed was increased to 900 rpm and water was added under mechanical stirring at 40 °C for 30 min.
(f) Distillation of acetone: The residual acetone was removed in rotavapor (Büchi, Flawil, Switzerland) at 50 °C under 300 mbar for 1 h.

The PUDs containing 0.04 wt % GO were synthesized by adding GO in different stages of their syntheses.

#### 2.2.1. PUD Obtained by Adding 0.04 wt % GO before Prepolymer Formation

0.04 wt % GO (1.27 g GO) was placed in polypropylene container and mixed with polyadipate of 1,4 butanediol in double orbital centrifuge Speed Mixer DAC 150.1 FVZ-K (Hauschild Engineering, Hamm, Germany) operating at 2000 rpm for 30 s. For removing the residual water, the mixture was heated at 75 °C for 30 min. The synthesis was carried out similarly to the one of PUD, but the polyol+GO mixture was added at the beginning of the synthesis for in-situ polymerization during prepolymer formation (Scheme 1). This PUD is named as PUD+0.04 wt % GO in polyol.

#### 2.2.2. PUD Obtained by Adding 0.04 wt % GO after Prepolymer Formation

0.04 wt % GO (1.27 g GO) was placed in polypropylene container and heated at 75 °C for 30 min for removing the residual water. Then, it was cooled down to 60 °C and 70.2 g acetone (the same amount used in the synthesis of PUD without GO) was added, placing the mixture in a double orbital centrifuge Speed Mixer operating at 2000 rpm for 150 s. The synthesis was carried out similarly to the one of PUD but the GO + acetone mixture was added after prepolymer synthesis for in-situ polymerization with the end NCO groups of the prepolymer (Scheme 1). This PUD is named as PUD+0.04 wt % GO after prepolymer.

#### 2.2.3. PUD Obtained by Adding 0.04 wt % GO in Water

0.04 wt % GO (1.27 g GO) and 120.7 g water were mixed in a glass container at 40 °C for 30 min. The synthesis was carried out similarly to the one of PUD, but the GO+water mixture was added after chain extension step for allowing the mixing of the GO with the polyurethane-urea chains by solvent mixing during the PUD particles formation (Scheme 1). This PUD is named as PUD+0.04 wt % GO in water.

Always, a 1.27 g GO slurry was added, and solid GO amounts between 0.048 and 0.052 wt % were experimentally determined in the PU films containing GO. 

**Figure 1:**
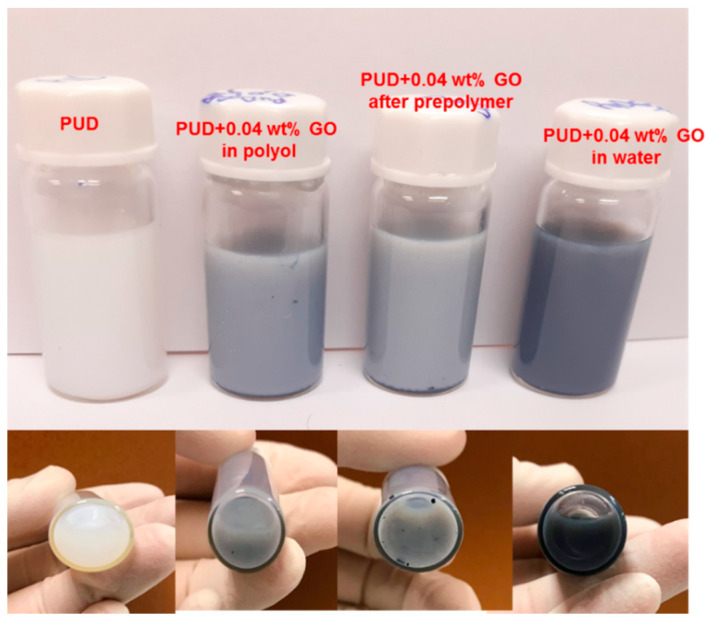
Appearance of the PUDs without and with 0.04 wt % GO.

Figure 1 shows the appearance of the PUDs without and with 0.04 wt % GO. After 1 month of PUD + 0.04 wt % GO after prepolymer dispersion synthesis, some precipitation of GO sheets into the bottle was found. 

Several properties were measured in solid polyurethane-urea (PU) films obtained by placing 12 g PUD or PUD + 0.04 wt % GO in square silicone mould of dimensions 10 cm × 10 cm, the water was removed inside a glass hood under atmospheric pressure and room temperature for one week.

Thinner PU films for stress-strain tests were prepared by placing 12 g PUD or PUD + 0.04 wt % GO on glass coated Teflon^®^ substrate of dimensions 12 mm × 24 mm. Three pieces of double-sided tape (3M, St. Paul, MN, USA) were placed over the sides of the mould for adjusting a thickness of 200 µm; once the PUD was spread over the mould, the water was left evaporating inside a glass hood under atmospheric pressure and room temperature for 24 h.

### 2.3. Experimental Techniques

#### 2.3.1. Characterization of the Polyurethane-Urea Dispersions (PUDs) without and with 0.04 wt % GO

##### Solids Content

The solids content of the GO slurry and the PUDs were determined in a DBS 60-3 thermo balance (Kern & Sohn GmbH, Balingen, Germany). About 0.5 g sample was heated at 105 °C for 15 min and then heated at 120 °C until constant mass. Three replicates for each sample were carried out and averaged.

##### pH Measurement

The pH values of the PUDs were measured at 25 °C in a pH-meter PC-501 (XS Instruments, Carpi, Italy) equipped with XC-PC510 electrode. Three replicates were measured and averaged.

##### Viscosity

The viscosities of the PUDs were measured at 25 °C in a DHR-2 rheometer (TA Instruments, New Castle, DE, USA) using coaxial cylindrical geometry according to DIN 53019 standard. The gap was set to 2 mm. The viscosities were measured as a function of the shear rate [36].

#### 2.3.2. Characterization of the Polyurethane-Urea (PU) Films without and with 0.04 wt % GO

##### Attenuated Total Reflectance Fourier Transform Infrared (ATR-IR) Spectroscopy

The ATR-IR spectra of the PU films were obtained in a Tensor 27 FT-IR spectrometer (Bruker Optik GmbH, Ettlinger, Germany) by using Golden Gate single reflection diamond ATR accessory. 64 scans with resolution of 4 cm^−1^ were recorded and averaged. ATR-IR spectra were normalized to the most intense band—C=O band at 1730 cm^−1^.

##### Raman Spectroscopy

The Raman spectra of the PU films were recorded in a Raman spectrometer Jasco NRS-5100 (Jasco, Madrid, Spain) by using an excitation wavelength of 532 nm (HeNe source), an exposure time of 40 s and a power of 0.7 mW. Raman spectra were repeated twice and were normalized to the most intense band—C-H band at 2927 cm^−1^.

##### Differential Scanning Calorimetry (DSC)

The DSC traces of the PU films were obtained in a TA DSC Q100 V6.2 Instrument (TA Instruments, New Castle, DE, USA). Hermetically closed aluminium pans containing a 10–15 mg sample were heated from −70 to 110 °C under nitrogen atmosphere (flow rate: 50 mL/min), the heating rate was 10 °C/min. Then a cooling run from 110 to −80 °C was carried out by using a cooling rate of 10 °C/min, and finally a second DSC heating run from −80 to 200 °C was carried out by using a heating rate of 10 °C/min. The glass transition temperatures (T_g_s) of the PU films were calculated at the inflexions of the second DSC heating traces.

##### Thermal Gravimetric Analysis (TGA)

The structure and thermal properties of the PU films were assessed in a TGA Q500 equipment (TA Instruments, New Castle, DE, USA). 10–15 mg PU film were placed in platinum crucible and heated under nitrogen (flow rate: 100 mL/min) from room temperature up to 800 °C, by using a heating rate of 10 °C/min.

##### X-Ray Diffraction (XRD)

The crystallinity of the PU films was determined by wide angle X-ray diffraction in a Bruker D8-Advance equipment (Bruker, Ettlingen, Germany), a voltage of 40 kV and the wavelength of copper kα (1.5418 Å) were used.

##### Plate-Plate Rheology

The rheological and viscoelastic properties of the PU films were measured in a DHR-2 rheometer (TA Instruments, New Castle, DE, USA) by using parallel plates (upper plate diameter = 20 mm) geometry. The gap used was 400 µm. The solid sample was placed on the bottom plate heated at 200 °C, then allows to melt for setting the gap and the measurements were carried out by decreasing the temperature from 200 to 25 °C in Peltier system, a cooling rate of 5 °C/min was used. The rheological experiments were performed in the region of linear viscoelasticity.

##### Stress-Strain Tests

The mechanical properties of the PU films were assessed by stress-strain tests made according to ISO 37 standard in an Instron 4411 universal testing machine (Instron, Buckinghamshire, UK) provided with mechanical extensometer, a pulling rate of 100 mm/min was used. Dog-bone test specimens of 0.05–0.13 mm thick and 25 mm length of the narrow portion were used and five replicates were measured and averaged. The tensile strength and elongation-at-break were measured at the points of the stress-strain curves were a sudden stress drop was produced.

##### Confocal Laser Microscopy

The dispersion of the GO sheets in the PU films was assessed in a Leica TCS SP2 microscope (Leica, Heidelberg, Germany). A drop of PUD was placed on glass microscope slide and then covered by a small glass cover slide, the PU films was obtained by drying at room temperature for 72 h, followed by heating at 40 °C for 8 h.

##### Water Contact Angle Measurements

The contact angle measurements were carried out at 21 °C by using bi-distilled and deionized water. The water contact angles were measured on the surfaces of the PU films in an ILMS goniometer (GBX Instruments, Bourg de Pèage, France). At least five water droplets of 4 µL were placed on different locations of each PU film surface, and the contact angle was calculated as the average of the values obtained.

#### 2.3.3. Adhesion Properties

##### T-peel Tests of Plasticized PVC/Polyurethane-Urea Dispersion/Plasticized PVC Joints

**Figure 2:**
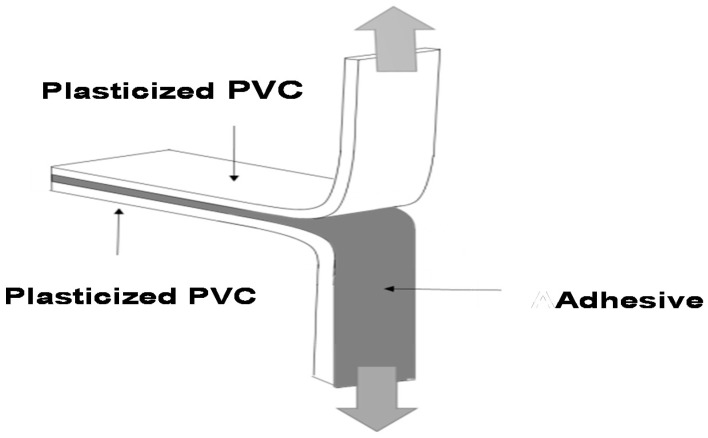
Scheme of the T-peel test of plasticized PVC/polyurethane-urea dispersion/plasticized PVC joints.

Adhesive strengths (under peeling stresses) of the joints made with polyurethane-urea dispersions were obtained from T-peel tests of solvent-wiped plasticized PVC/polyurethane-urea dispersion/solvent-wiped plasticized PVC joints (Figure 2) [36]. The plasticized poly(vinyl chloride)—PVC—test samples were methyl ethyl ketone wiped. 0.90 g dispersion was applied by brush to each PVC strip allowing the water evaporates at 25 °C for 1 h and the adhesive films were heated suddenly at 80 °C for 10 s under infrared radiation (reactivation process). The PVC strips were immediately placed in contact, applying a pressure of 0.8 MPa for 10 s. The T-peel strength was measured 1 and 72 h after joint formation in an Instron 4411 universal testing machine (Instron Ltd., Buckinghamshire, UK), a crosshead speed of 100 mm/min was used. Three replicates were measured and averaged. The loci of failure of the joints were assessed by visual inspection.

##### Single Lap-Shear Test of Stainless Steel 304/Polyurethane-Urea Dispersion/Stainless Steel 304 Joints

**Figure 3:**
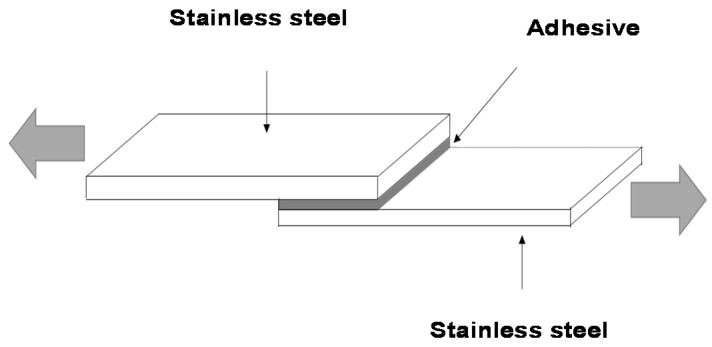
Scheme of the single lap-shear test of stainless steel 304/polyurethane-urea dispersion/stainless steel 304 joints.

The adhesion of the polyurethane-urea dispersions under shear stresses was carried out by single lap-shear tests of stainless steel 304/polyurethane-urea dispersion/stainless steel 304 joints (Figure 3) [36]. Stainless steel 304 specimens were mechanically abraded and wiped with isopropanol. 0.2 g dispersion was placed on 1.5 cm × 3 cm surface of one of the stainless steel specimens, leaving it evaporates at room temperature for 15 min; then, the other stainless steel specimen was placed on top, applying a pressure of 0.16 kPa at room temperature for 5 days. The single lap-shear tests were carried out in an Instron 4411 universal testing machine (Instron Ltd., Buckinghamshire, UK), a pulling rate of 10 mm/min was used. At least three replicates for each adhesive joint were determined and averaged. The loci of failure of the joints were assessed by visual inspection.

## 3. Results and Discussion

### 3.1. Characterization of the Graphene Oxide (GO)

The experimental solid GO content in the GO slurry was 3.1 ± 0.6 wt %. The characterization of GO has been carried out elsewhere [36] and, in this study, only its main features are summarized. The chemical composition of the GO surface assessed by XPS corresponds to 64 at. % carbon, 35 at. % oxygen and 1 at. % nitrogen, and the curve fitting of the high resolution C1s spectrum shows the existence of C–O in phenolic groups mainly (31 at. %), and minor contributions of C=O (5 at. %) and O-C=O (2 at. %) groups [36], in agreement with previous study [37]. On the other hand, the X-ray diffractogram of GO shows three main diffraction peaks at 2Ө values of 9.55°—typical of GO, and 18.30 and 25.35°—due to reduced GO [38]. Finally, the GO morphology corresponds to 4-10 stacked graphene layers of less than 20 nm thick and 2–5 µm long [36].

### 3.2. Characterization of the PUDs

GO was added in three different stages of the PUD synthesis for dispersing differently the GO sheets in the polyurethane-urea chains by creating chemical (in-situ polymerization) or physical (solvent mixing) interactions between them.

**Figure 4:**
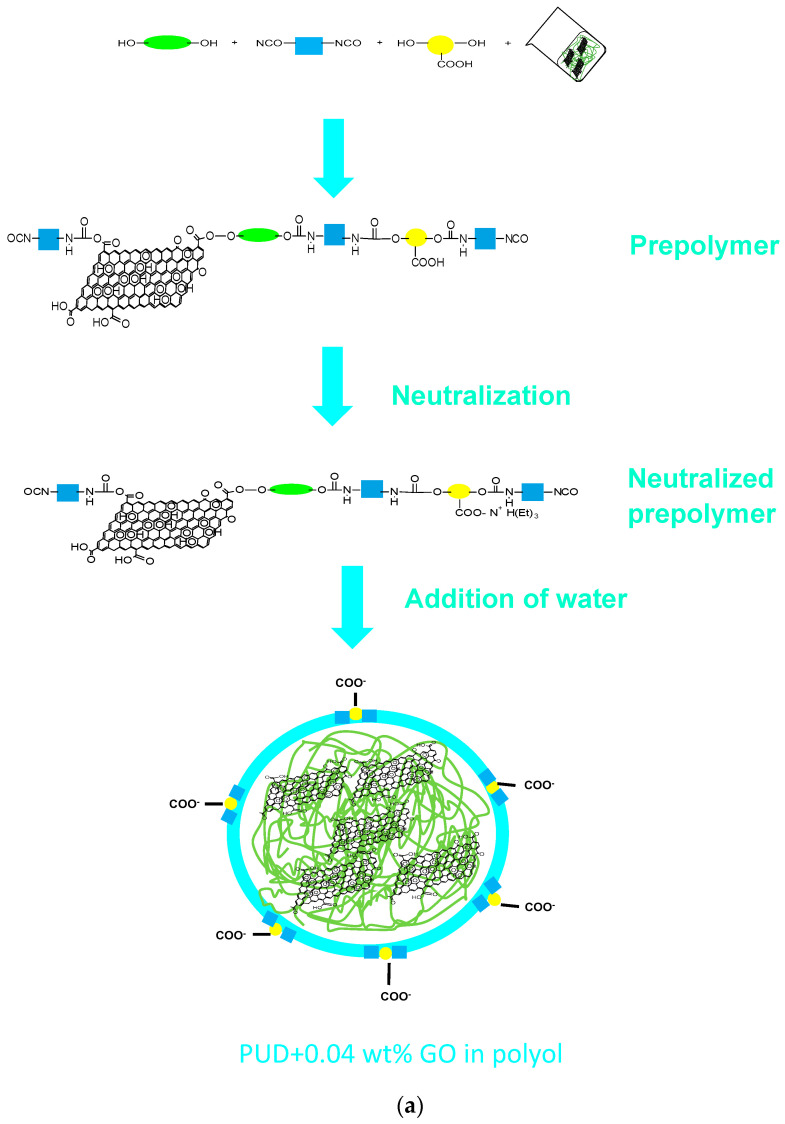

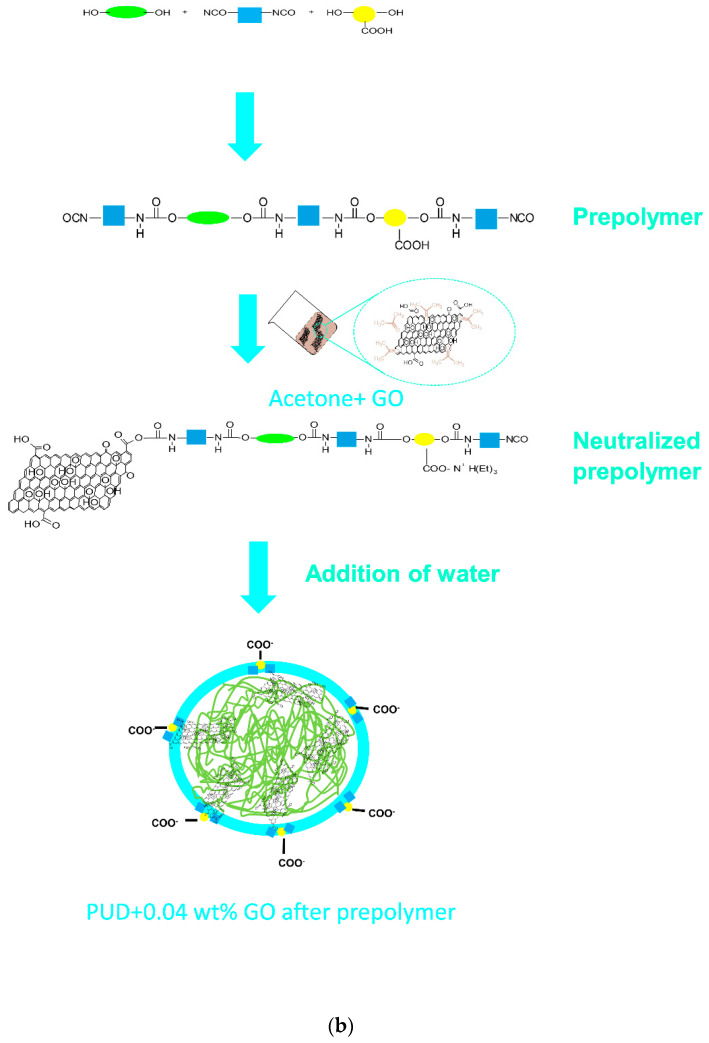

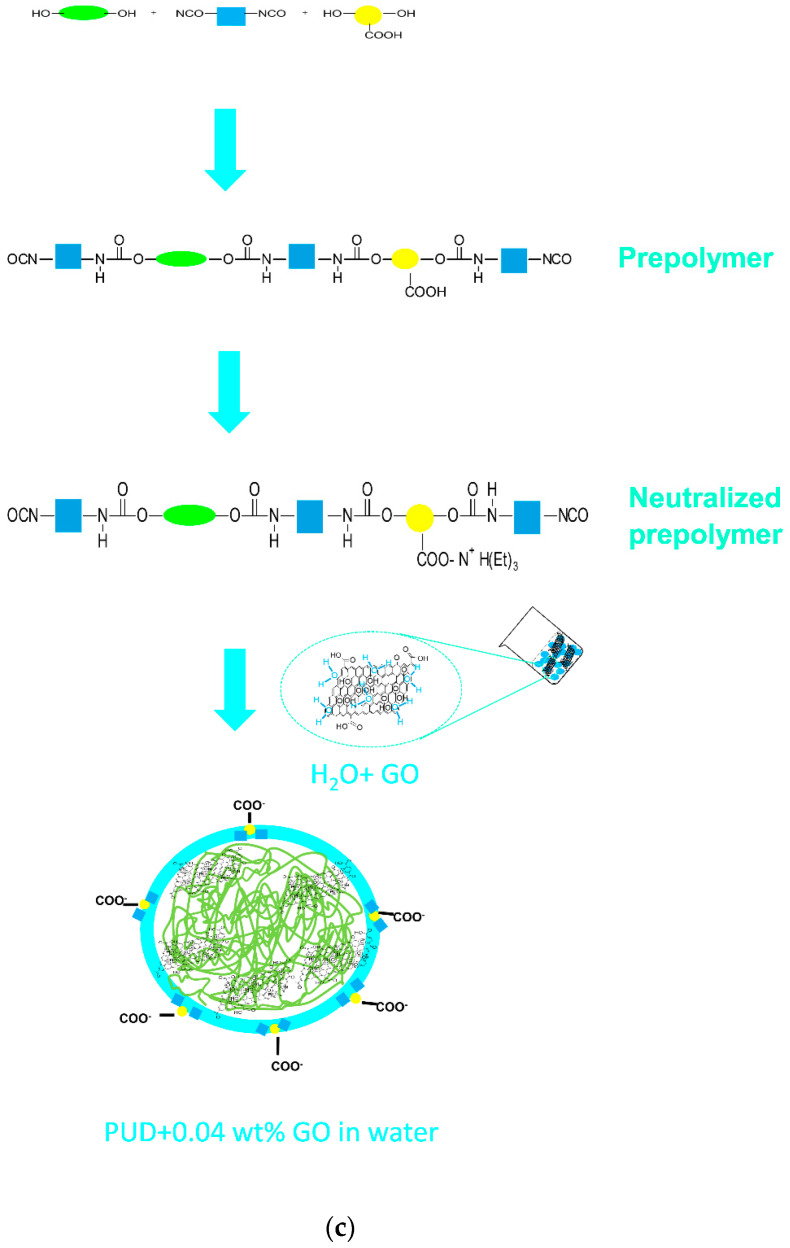
(**a**) Scheme of the interactions of the GO sheets in the prepolymer and structure of PUD+0.04 wt % in polyol; (**b**) Scheme of the interaction of the GO sheets with the NCO groups of the prepolymer and structure of PUD+0.04 wt % after prepolymer; (**c**) Scheme of the trapping of the GO sheets between the polyurethane-urea chains and structure of PUD+0.04 wt % in water.

Addition of GO before prepolymer formation (PUD+0.04 wt % GO in polyol). The functional groups on the edges of the GO sheets should react with the NCO groups of the isocyanate (in-situ polymerization) creating covalent bonds. Therefore, the prepolymer will have covalently bonded GO sheets all along its lineal polymeric structure and they will be trapped between the polyurethane chains during the addition of water at the end of the synthesis (Figure 4a).

Addition of GO after prepolymer formation (PUD+0.04 wt % GO after prepolymer). The NCO groups at the end of the prepolymer chain will react with the functional groups of the GO sheets creating covalent bonds (in-situ polymerization). With respect to the addition of GO before prepolymer formation, the separation between the covalently bonded GO sheets to the prepolymer is longer and more regularly distributed (Figure 4b).

Addition of GO during water addition stage (PUD+0.04 wt % GO in water). Once the polyurethane-urea chains are formed and dissolved in acetone, the formation of PUD particles is produced by adding water. GO is added in water for physical trapping of the GO sheets between the polyurethane-urea chains (solvent mixing), and, therefore, no chemical interactions are produced (Figure 4c). With this method, GO is expected to act as a nanofiller for polyurethane-urea composite.

The particle size distribution of the PUD was narrow and its mean particle size was 45 nm. The solids contents and the pH of the PUDs without and with 0.04 wt % GO are given in Table 1. The solids contents of the PUDs are near 39 wt % except for PUD+0.04 wt % GO in polyol, and the pH values are near 9, the addition of GO does not change significantly the pH.

**Figure 5:**
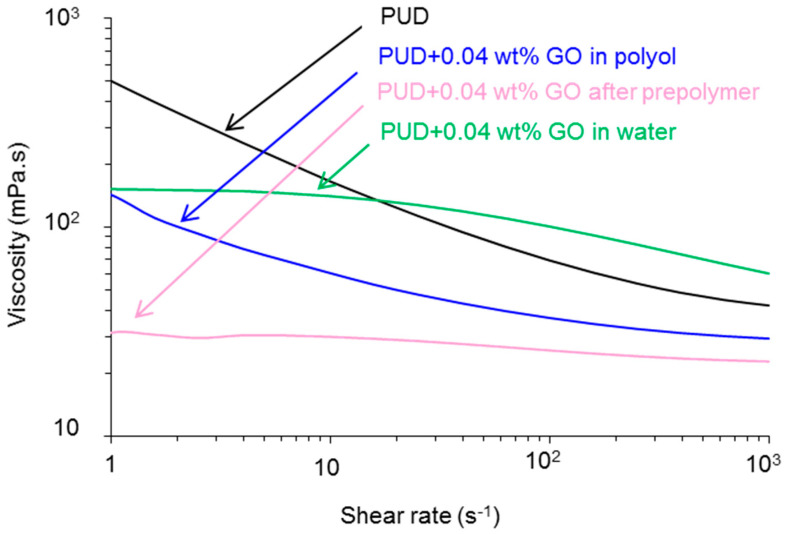
Variation of the viscosity of PUD and PUD + 0.04 wt % GO as a function of the shear rate.

The variation of the viscosity at 25 °C of PUDs without and with 0.04 wt % GO as a function of the shear rate is given in Figure 5. The viscosity of PUD without GO decreases by increasing the shear rate, i.e., non-Newtonian rheological behavior—shear thinning—is shown. The viscosity of PUD+0.04 wt % GO in water measured at 700 s^−1^ is higher than the one of PUD (Table 2), a typical behavior of filled polyurethane in which the filler is intercalated among the polymer chains increasing the interactions between them [15]; furthermore, the shear thinning is much less marked. Unexpectedly, the viscosities of the PUDs containing GO added before and after prepolymer formation are lower than the one of PUD (Table 2). The viscosity of the PUDs containing GO can be ascribed to the extent of the ionic interactions between the particles inside which the covalent interactions between GO and the polyurethane-urea chains lead to changes on the particles surfaces decreasing the interactions between them. The variations in shear thinning of the PUDs have been coarsely estimated by the “pseudoplasticity index” which is obtained as the ratio of viscosities measured at shear rates of 1 and 700 s^−1^. The more shear thinning of the PUD, the higher pseudoplasticity index value. The highest pseudoplasticity index corresponds to PUD (Table 2) in which the ionic interactions between the particles are stronger, and the lowest one corresponds to PUD+0.04 wt % after prepolymer.

In a previous study [39], the non-Newtonian behavior of GO nanocomposites has been mathematically modelled using the power law for viscosity, the plateau values for lower (η_0_) and upper (η_∞_) Newtonian viscosities have been obtained. Furthermore, that model allows the calculation of τ and m parameters; τ parameter corresponds to the reciprocal of the shear rate at which the viscosity is η_0_ and m parameter is related to the power low index, n (m = 1 − n). The results of the application of the model to the PUDs are given in Table 3 in which the same trends than in Table 2 are obtained. Thus, at very low shear rates, the viscosities of the PUDs containing GO mixtures are considerably lower than that of PUD, the lowest η_0_ corresponds to PUD-0.04 wt % GO after prepolymer. However, at higher shear rate values, the highest η_∞_ corresponds to PUD+0.04 wt % GO in water as expected for a filled polyurethane, but the η_∞_ values for the other PUDs containing GO are lower than that of PUD. The values of the parameter m for the PUDs containing GO, are very similar and significantly lower than for PUD, and the values of τ are also lower in the PUDs containing GO, indicating that the shear thinning behavior starts at higher shear rate values, especially for PUD+0.04 wt % GO after prepolymer.

### 3.3. Characterization of the PU Films

**Figure 6:**
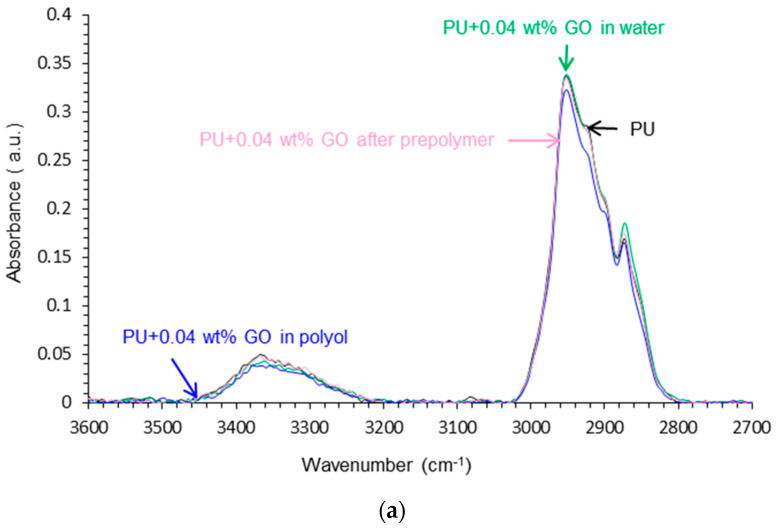

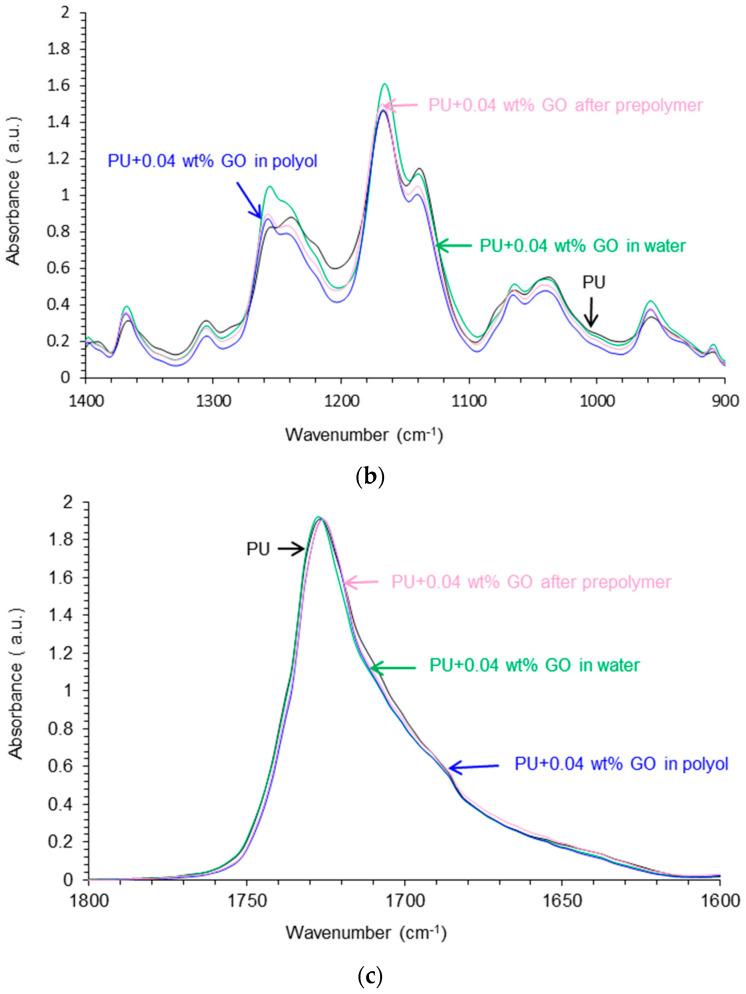
(**a**) 3600–2700 cm^−1^ region of the ATR-IR spectra of PU and PU + 0.04 wt % GO films; (**b**) 1400–900 cm^−1^ region of the ATR-IR spectra of PU and PU + 0.04 wt % GO films; (**c**) Carbonyl region (1800–1600 cm^−1^) of the ATR-IR spectra of PU and PU + 0.04 wt % GO films.

The ATR-IR spectra of the polyurethane-urea films without (PU) and with 0.04 wt % GO (PU + 0.04 wt % GO) are given in Figure 6a–c. The bands due to the hard segments appear at 3367 cm^−1^ (N-H stretching), 1727 cm^−1^ (C=O stretching), and 1532 cm^−1^ (C-N stretching); the bands of the soft segments correspond to C-H stretching at 2873 and 2952 cm^−1^, CH_2_ bending at 1461 cm^−1^, and several C-O bands of the polyester polyol at 1239, 1140, 1166, 1065 and 958 and 735 cm^−1^. The addition of 0.04 wt % GO does not produce new bands in the ATR-IR spectra of the PU films but some changes of the wavenumber of some absorption bands are observed, these changes can be associated to a structural reorganization [35]. Thus, the N-H band at 3367 cm^−1^ in the PU film without GO is displaced to 3354 cm^−1^ in PU+0.04 wt % GO in polyol, indicating the existence of interactions between the functional groups on the GO sheets and the hard segments of the polyurethane-urea (Figure 6a). On the other hand, according to Figure 6b, the addition of GO displaces the C-O band from 1239 cm^−1^ (PU film without GO) to 1256–1257 cm^−1^, indicating different interactions between the soft segments caused by the GO sheets; furthermore, the intensities of the bands at 1305 and 1370 cm^−1^ due to the absorption of the carboxylic acid salt of DMPA hard segments vary differently (Figure 6b) because the GO-polyurethane urea interactions differ depending on the stage of addition of GO during the synthesis, the lowest intensity is obtained in PU+0.04 wt% GO in polyol. 

**Figure 7:**
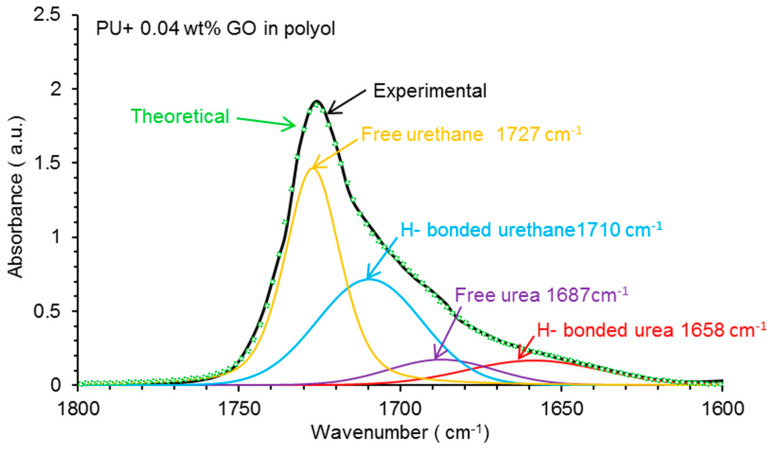
Curve fitting of the carbonyl region (1800–1600 cm^−1^) of the ATR-IR spectrum of PU+0.04 wt % GO in polyol.

Figure 6c shows the carbonyl region (1800–1600 cm^−1^) of the ATR-IR spectra of the PU and PU + 0.04 wt % GO films and differences in the regions of the associated by hydrogen bond urethanes and the urea groups are noticed. In order to quantify these differences, the curve fitting of the carbonyl region (1800–1600 cm^−1^) of the ATR-IR spectra of the PU films have been carried out, 99 % Gaussian and 1 % Lorentz distributions were used in OPUS 6 5 FTIR software (Bruker). The curve fitting of the carbonyl region of the ATR-IR spectrum of PU+0.04 wt % GO in the polyol is given, as typical example, in Figure 7 in which four contributions due to free urethane (1727 cm^−1^), associated by hydrogen bond urethane (1710 cm^−1^), free urea (1687 cm^−1^), and associated by hydrogen bond urea (1658 cm^−1^) groups are found. Table 4 shows the percentages of urethane and urea groups in the PU films with and without 0.04 wt % GO. The addition of GO decreases the percentages of free urethane and free urea groups, this is an evidence of the interaction between the hard segments and the functional groups on the GO sheets. On the other hand, the percentage of associated by hydrogen bond urethane groups increases noticeably in the PU films containing GO likely due to the change of the degree of micro-phase separation induced by GO. PU+0.04 wt % GO in polyol shows the lowest free urea percentage, and PU and PU+0.04 wt % GO after prepolymer exhibit the lowest free urethane percentage; however, the differences in the wavenumber at which the different species appear are not significant.

**Figure 8:**
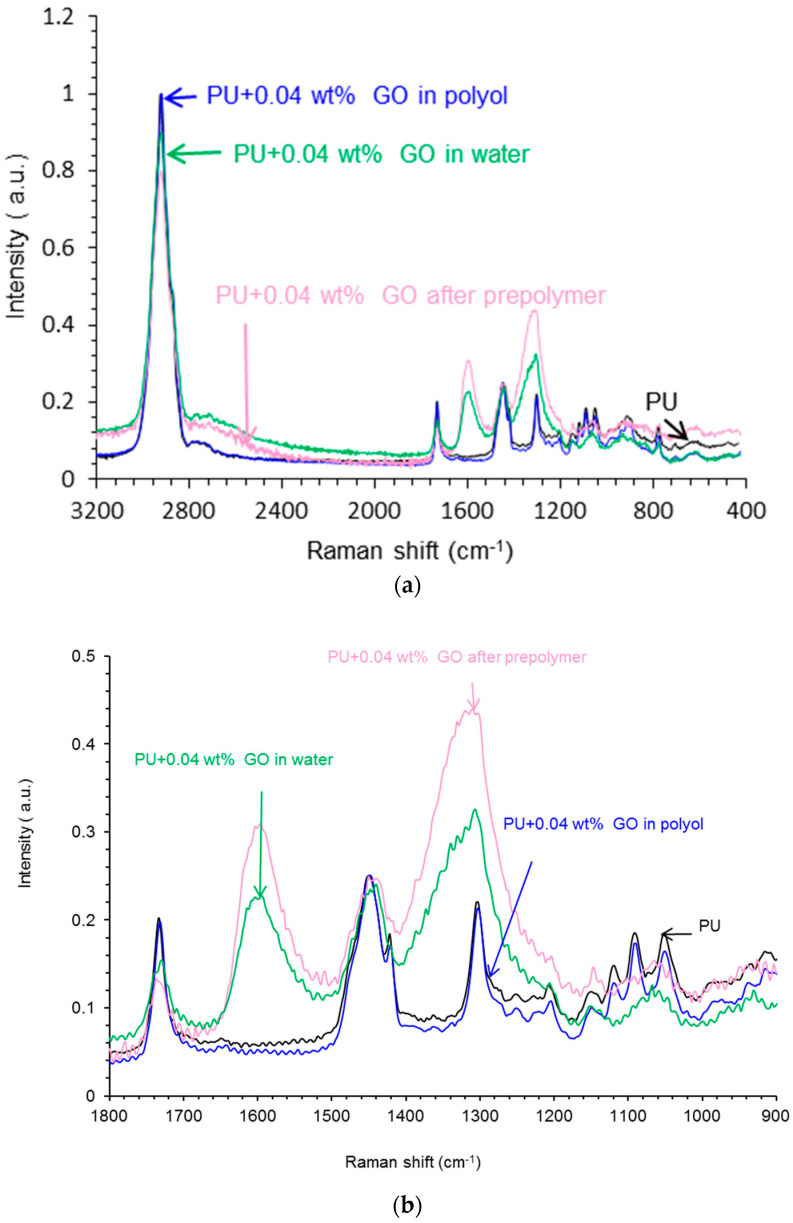
(**a**) Raman spectra of PU films; (**b**) 1800–600 cm^−1^ region of the Raman spectra of PU films.

Raman is more sensitive than IR spectroscopy for evidencing the interactions between GO and the polyurethane-urea chains. Figure 8a shows the Raman spectra of PU films. The C=O band of the hard segments appears at 1733 cm^−1^, and the bands of the soft segments are evidenced at 2927 cm^−1^ (C-H) and 1449–1450 cm^−1^ (CH_2_), and several C-O bands of the polyester polyol at 1255–1257, 1130, 1164, 1036, 1066 and 948 cm^−1^ can be distinguished. The addition of GO decreases the intensity of the C-H band at 2927 cm^−1^ and new bands at 1342 and 1598 cm^−1^ appear (Figure 8b), these Raman shifts correspond to the D and G bands of Raman spectrum of GO, the existence of interactions between the polyurethane-urea chains and GO is evidenced. However, the Raman spectra of PU and PU+0.04 wt % GO in polyol are somewhat similar.

**Figure 9:**
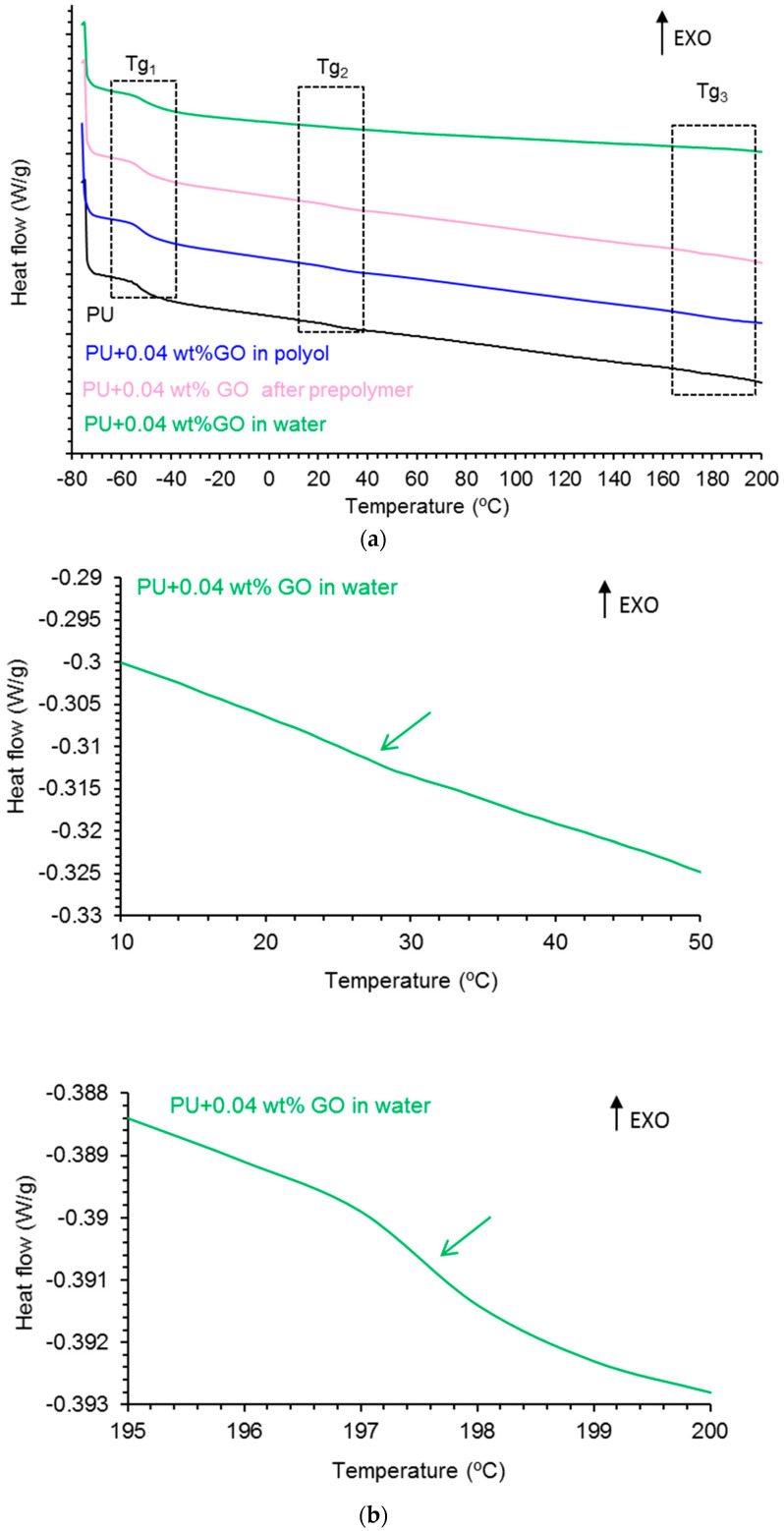
(**a**) DSC traces of PU films. Second heating run; (**b**) Expanded regions of DSC trace of PU+0.04 wt % GO in water. Second heating run.

The DSC traces of PU films (Figure 9a) show similar features, i.e., the glass transition temperatures of the soft segments (T_g1_ and T_g2_) at −49 and 30 °C, and the one of the hard segments (T_g3_) at 198 °C (Table 5). The expanded regions of the glass transition regions of the soft and hard segments in the DSC trace of PU+0.04 wt % GO in water, taken as typical example, are shown in Figure 9b. The addition of 0.04 wt % GO shifts slightly the T_g_ of the soft segments to lower values (Table 5), and the T_g_ of the hard segments also decreases—mainly in PU+0.04 wt % GO after prepolymer -, this suggests that the addition of GO changes the mobility of the polyurethane-urea chains and the degree of micro-phase separation between the hard and soft segments. This change could be mainly attributed to the gathering of GO sheets in the vicinity of the hard segments in the polyurethane via covalent and/or hydrogen bond interactions [40].

**Figure 10:**
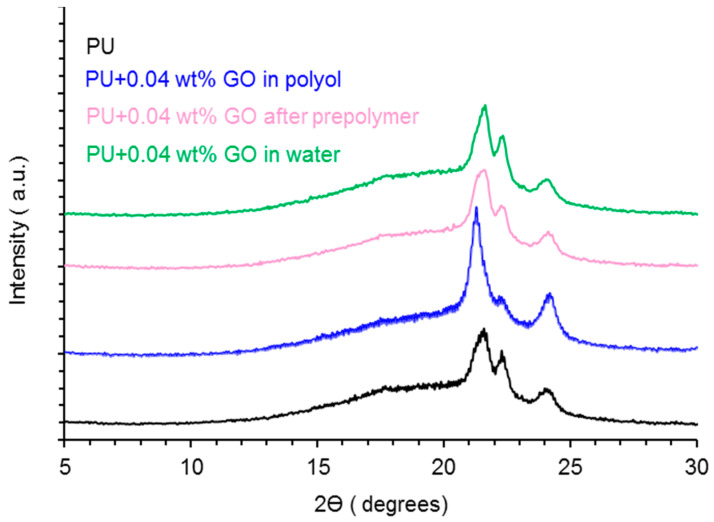
X-ray diffractograms of PU films.

The crystallinity of PU films was determined by X-ray diffraction. The crystallites in PU films are due to the interactions between the polyester soft segments which decrease their motion and favor the micro-phase separation. Figure 10 shows the X-ray diffractograms of PU films in which the characteristic diffraction peak of GO at 2Ө of 9.55° is not observed, indicating the interaction with the polymer chains. Additionally, three diffractions peaks can be distinguished at 2Ө values of 21.3–21.7°, 21.9–22.4° and 24.0–24.2°, all these correspond to the crystalline structure of the polyester polyol. The diffraction peaks of the PU films appear at the same 2Ө values except in PU+0.04 wt % GO in polyol, in which the diffraction peaks are displaced to lower 2Ө values indicating increased interlayer spacing of the GO sheets due to the interactions with the polyurethane-urea chains, in agreement with previous study in GO-polyurethane composites [41]. On the other hand, the intensities of the diffraction peaks of the PU films are similar except in PU+0.04 wt % GO in polyol in which the intensities are higher because of stronger interactions between the soft segments caused by the interactions between GO and polyurethane-urea.

**Figure 11:**
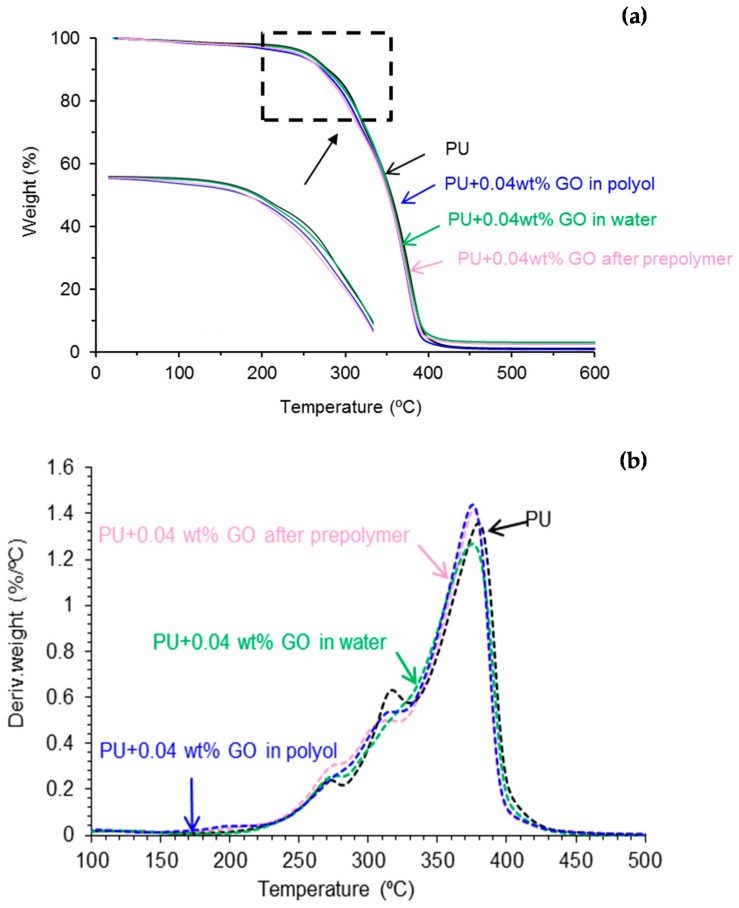
Variation of (**a**) the weight and (**b**) the derivative of the weight of PU films as a function of the temperature. TGA experiment.

Figure 11a shows that the thermal stabilities of the PU films decrease by adding 0.04 wt % GO, except in PU+0.04 wt % GO in water, and they are quantified by the temperatures at which 5 (T_5%_) and 50 (T_50%_) wt % are lost. According to Table 6, the value of T_5%_ decreases in the PU films containing GO added before and after prepolymer formation, and they are similar in PU and PU+0.04 wt % GO in water. Therefore, the covalent interactions between the GO surface groups and the polyurethane-urea chains change the degree of micro-phase separation in PU+0.04 wt % GO in polyol and PU+0.04 wt % GO after prepolymer, but not when physical GO-polyurethane-urea interactions are produced (PU+0.04 wt % GO in water). Some literature has established the improved thermal stability of polyurethane composites containing graphene derivatives [13,19,23,27], but the opposite trend is found in this study; this can be due to the small GO amount added and to the dominant effect of the GO sheets in changing the structure of the hard and soft domains.

The structural changes in PU films caused by adding 0.04 wt % GO can be assessed from the thermal decompositions of the derivative TGA plots (Figure 11b). PU and PU+0.04 wt % GO in water show a small thermal decomposition (2–4 wt %) at 114–126 °C due to retained moisture (Table 7). The PU film without GO shows three thermal decompositions due to the urethane hard domains (270 °C), urea hard domains (315 °C) and soft domains (380 °C), the higher weight loss corresponds to the soft domains. The addition of GO maintains the temperatures and weight losses of the three thermal decompositions in the PU films, but the decomposition temperature of the urethane hard domains is higher in PU+0.04 wt % GO in polyol, and the one of the urea hard domains is lower PU+0.04 wt % GO after prepolymer; furthermore, both PU+0.04 wt % GO in polyol and PU+0.04 wt % GO after prepolymer show an additional decomposition (4–5 wt %) at 196-211 °C (Table 7), which can be ascribed to the thermal rupture of the GO-polyurethane-urea interactions. Therefore, the addition of GO before or after prepolymer formation produces more noticeable changes in the degree of micro-phase separation than in PU+0.04 wt % GO in water.

**Figure 12:**
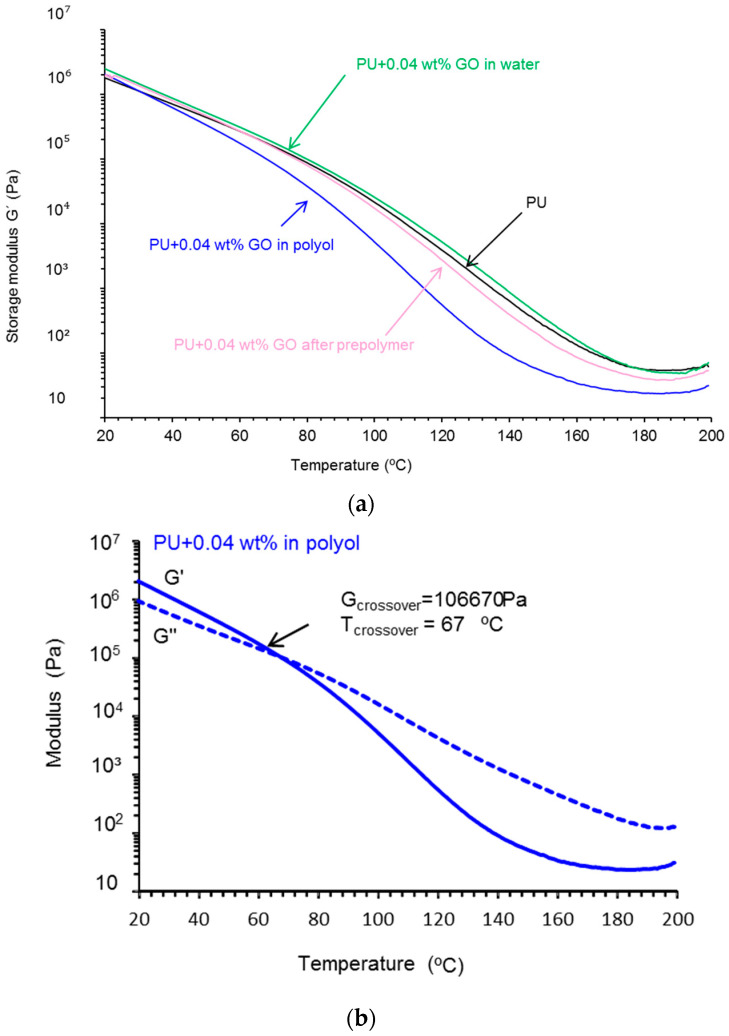
(**a**) Variation of the storage modulus (G′) as a function of the temperature for PU films. Plate-plate rheology experiments; (**b**) Variation of the storage (G′) and loss (G″) moduli of PU+0.04 wt % GO in the polyol as a function of the temperature. Plate-plate rheology experiments.

The rheological and viscoelastic properties of the PU films are also modified by adding 0.04 wt % GO. Figure 12a shows the variation of the storage modulus (G′) as a function of the temperature of PU films. The addition of 0.04 wt % GO in the polyol decreases the storage modulus of the PU film, more markedly by increasing the temperature, but G′ values are somewhat similar in the other PU films, this indicates a different structure in PU+0.04 wt % GO in polyol than in the rest.

Figure 12b shows, as a typical example, the variation of the storage (G′) and loss (G″) moduli as a function of the temperature for PU+0.04 wt % GO in polyol. The viscous rheological regime is dominant above 81 °C in PU+0.04 wt % GO in polyol and the elastic rheological regime is dominant below 81 °C, i.e., there is a crossing of G′ and G″ at 81 °C. The viscoelastic properties of the PU films are mainly determined by their soft segments and degree of micro-phase separation, and, therefore, the values of temperature (T_cross-over_) and modulus (G_cross-over_) at the crossing of G′ and G″ will change [42]. According to Table 8, the G_cross-over_ and T_cross-over_ values of all PU films are somewhat similar, except in PU+0.04 wt % GO in polyol that shows higher G_cross-over_ and lower T_cross-over_ values. Therefore, the addition of GO before prepolymer formation causes more noticeable structural changes and shows more marked degree of micro-phase separation of the polyurethane-urea than when GO is added after prepolymer formation or in water.

**Figure 13:**
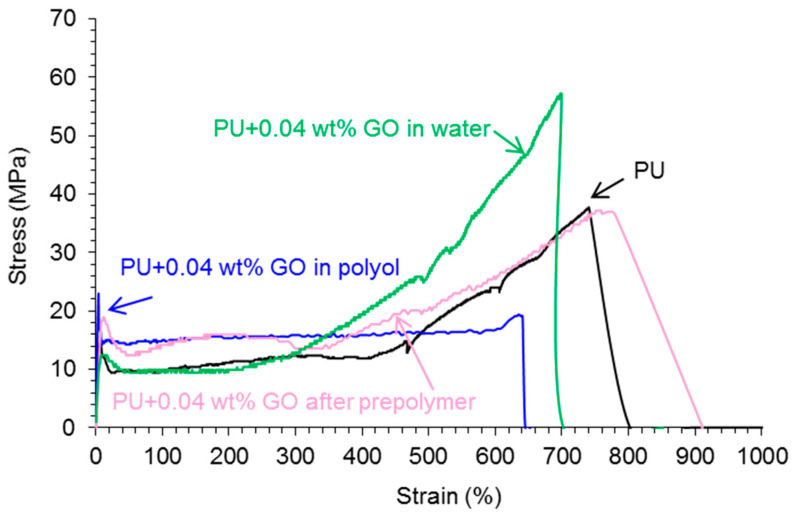
Stress-strain curves of PU films.

The mechanical properties of PU films were assessed by stress-strain tests. According to Figure 13, all stress-strain curves show the elastic region followed by a noticeable yield point and the long region of the plastic deformation, this one is less marked in PU+0.04 wt % GO in polyol. The highest yield stress and lower yield strain corresponds to PU+0.04 wt % GO in polyol, but this PU film exhibits lower tensile strength and elongation-at-break than the rest (Table 9). The highest tensile strength without decrease of the elongation-at-break corresponds to PU+0.04 wt % GO in water. Therefore, the mechanical properties of the PU films are affected differently by adding GO in different stages of the synthesis of the PUDs.

**Figure 14:**
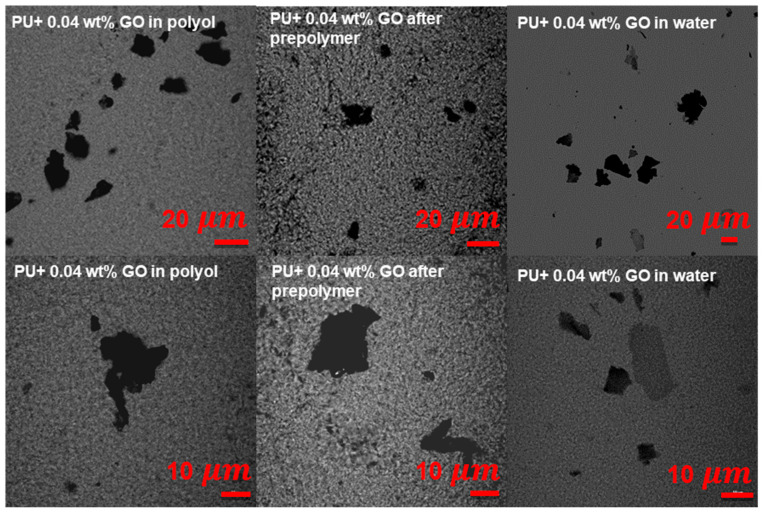
Confocal laser micrographs of PU films at different magnifications.

The dispersion of the GO particles in the polyurethane matrix in the PU films was assessed by confocal laser microscopy. Figure 14 shows that the GO sheets are well dispersed into the polyurethane matrix without the existence of agglomerates, and particles of about 5 and 15 µm length can be distinguished, irrespective of the stage of the synthesis in which GO is added.

Because of the differing polarity of the polyurethane-urea and GO, the wettability of the PU films may change by adding 0.04 wt % GO. The wettability of the PU films was assessed by water contact angle measurements. Table 10 shows that the lowest water contact angle value corresponds to the PU film without GO and the highest value is found in PU+0.04 wt % GO in water. Depending on the stage in which GO is added during the synthesis, the water contact angle value on the PU film changes differently because of the different degree of micro-phase separation on the surface.

### 3.4. Adhesion Properties of the PUDs

The adhesion of the PUDs was determined by T-peel test and single lap-shear tests.

Table 11 shows the values of the T-peel strength values of the plasticized PVC/PUD/plasticized PVC joints measured 1 and 72 h after joints formation. After 1 h of joint formation (immediate peel strength), the T-peel strength increases in the joints made with all PUDs containing GO. The T-peel strength increases by increasing the time after joint formation and, after 72 h, the T-peel strengths in the joints made with all PUDs containing GO are higher than in the one made with the PU film without GO. The highest T-peel value is obtained in the joint made with PUD+0.04 wt % GO in polyol likely due to its particular structure, thermal and viscoelastic properties caused by the existence of more net GO-polyurethane-urea interactions. Furthermore, a significantly higher T-peel strength is obtained in the joint made with PU+0.04 wt % GO after prepolymer, the lower adhesion corresponds to the one made with PU+0.04 wt % GO in water in which physical interactions between the GO sheets and the polymeric chains can be expected.

Table 12 shows the single lap-shear strength values of stainless steel 304/PUD/stainless steel 304 joints obtained after 5 days of joint formation. Whereas the lap-shear strength of the joint made with the PUD without GO is somewhat low, an increase in the single lap-shear strength is found in the joints made with all PUDs containing GO, the lap-shear strength is higher in the joint made with PU+ 0.04 wt % GO in water which mechanical properties are the highest. Always an adhesion failure is obtained.

## 4. Conclusions

PUDs have been synthesized by adding 0.04 wt % GO before and after prepolymer formation, and during water addition stage. The addition of GO before prepolymer formation more efficiently changed the polyurethane-urea structure, i.e., the covalently bonded GO sheets disturbed the interactions between the hard segments causing lower percentage of free urethane groups, higher crystallinity, lower storage modulus, higher yield stress, and higher T-peel strength. On the other hand, the addition of GO in water produced physical interactions with the polyurethane-urea chains, i.e., GO was acting as a nanofiller, which justified the improved mechanical properties and high lap-shear strength. Therefore, the addition of GO in different stages of the synthesis of the PUDs determined differently their structure, viscoelastic, and adhesion properties.

The addition of 0.04 wt % GO decreased the viscosity of the PUD, and the extent of shear thinning was much less marked, PUD+0.04 wt % GO in water is an exception. Furthermore, the addition of GO before prepolymer formation produced interactions between GO and polyurethane-urea causing changes of the degree of micro-phase separation between the hard and soft segments, which is evidenced by a decrease of the percentages of free urethane and free urea groups and a decrease of the one of associated urethane groups. The GO-polyurethane-urea interactions in the PU films containing GO added before and after prepolymer formation were also evidenced by the decrease of the T_g_ values of the soft and hard segments, the absence of the diffraction peak of GO at 2Ө value of 9.55° was not observed, and the additional decomposition (4–5 wt %) at 196–211 °C.

The GO sheets were well dispersed into the polyurethane matrix without the existence of agglomerates, and GO sheets of about 5 and 15 µm length can be distinguished, irrespective of the stage of the synthesis in which GO was added. Moreover, the highest water contact angle value was found in PU+0.04 wt % GO in water.

The T-peel strength increased in the joints made with all PUDs containing GO, the highest T-peel strength, was obtained in the joint made with PUD+0.04 wt % GO in polyol likely due to the existence of more net covalent GO-polyurethane-urea interactions. Furthermore, a significantly high T-peel strength was obtained in the joint made with PU+0.04 wt % GO after prepolymer, and the lowest adhesion corresponds to the one made with PU+0.04 wt % GO in water in which GO-polyurethane-urea physical interactions can be expected. On the other hand, an increase of the single lap-shear strength was found in the joints made with all PUDs containing GO, and the lap-shear strength was higher in the joint made with PU+0.04 wt % GO in water which mechanical properties were the highest.

## Figures and Tables

**Table 1:** Solids contents and pH values of PUD and PUD + 0.04 wt % GO dispersions.

Polyurethane-Urea Dispersion	Solids Content (wt %)	pH
PUD	38.6 ± 0.6	9.5 ± 0.2
PUD+0.04 wt % GO in polyol	36.8 ± 0.7	9.2 ± 0.2
PUD+0.04 wt % GO after prepolymer	39.1 ± 0.7	9.4 ± 0.2
PUD+0.04 wt % GO in water	39.0 ± 0.4	9.1 ± 0.0

**Table 2:** Viscosities at 25 °C at different shear rates and pseudoplasticity index of PUDs.

Polyurethane-Urea Dispersion	Viscosity at 1 s^−1^ (mPa·s)	Viscosity at 700 s^−1^ (mPa·s)	Pseudoplasticity Index *
PUD	505	42	12.0
PUD+0.04 wt % GO in polyol	142	30	4.7
PUD+0.04 wt % GO after prepolymer	31	23	1.3
PUD+0.04 wt % GO in water	150	60	2.5

* Pseudoplasticity index = Viscosity at 1 s^−1^/Viscosity at 700 s^−1^.

**Table 3:** Rheological parameters of PUDs obtained from the power law of viscosity modelling [39].

Polyurethane-Urea Dispersion	η_0_ (mPa·s)	η_∞_ (mPa·s)	τ (s)	m
PUD	1520	42	8.1	0.0240
PUD+0.04 wt % GO in polyol	360	29	2.4	0.0003
PUD+0.04 wt % GO after prepolymer	37	22	0.5	0.0002
PUD+0.04 wt % GO in water	170	59	2.6	0.0006

**Table 4:** Relative contributions of the free and associated urethane and urea groups in PU films. Curve fitting of the carbonyl region of the ATR-IR spectra.

Wavenumber (cm^−1^)	Relative Contribution of Species (%)			
	PU	PU+0.04 wt % GO in Polyol	PU+0.04 wt % GO after Prepolymer	PU+0.04 wt % GO in Water
1727 (free urethane)	58	45	38	42
1711–1709 (H-bonded urethane)	9	35	33	38
1693–1687 (free urea)	22	9	14	12
1658–1652 (H-bonded urea)	11	11	15	8

**Table 5:** Some thermal properties obtained from DSC traces of PU films. Second heating run.

Polyurethane-Urea Film	T_g1_ (°C)	T_g2_ (°C)	T_g3_ (°C)
PU	−49	30	198
PU+0.04 wt % GO in polyol	−52	28	195
PU+0.04 wt % GO after prepolymer	−52	27	174
PU+0.04 wt % GO in water	−52	26	197

**Table 6:** Values of temperatures at which 5 (T_5%_) and 50 (T_50%_) wt % are lost in PU films. TGA experiments.

Polyurethane-Urea Film	T_5%_ (°C)	T_50%_ (°C)
PU	255	356
PU+0.04 wt % GO in polyol	239	352
PU+0.04 wt % GO after prepolymer	244	352
PU+0.04 wt % GO in water	253	354

**Table 7:** Some data obtained from DTGA plots of PU films. At the end of TGA experiment, all PU films exhibit a residue of 2 wt %.

Polyurethane Urea Film	T_1_ (°C)	Weight Loss_1_ (%)	T_2_ (°C)	Weight Loss_2_ (%)	T_3_ (°C)	Weight Loss_3_ (%)	T_4_ (°C)	Weight Loss_4_ (%)
PU	114	2	270	10	315	24	380	62
PU+0.04 wt % GO in polyol	196	4	275	10	312	21	376	63
PU+0.04 wt % GO after prepolymer	211	5	271	11	304	18	377	64
PU+0.04 wt % GO in water	126	4	270	10	312	21	377	63

**Table 8:** Values of the temperature (T_cross-over_) and modulus (G_cross-over_) at the cross-over of the storage and loss moduli of PU films. Plate-plate rheology experiments.

Polyurethane-Urea Film	T _cross-over_ (°C)	G_cross-over_ (kPa)
PU	81	78.5
PU+0.04 wt % GO in polyol	67	106.7
PU+0.04 wt % GO after prepolymer	79	81.0
PU+0.04 wt % GO in water	83	80.8

**Table 9:** Some mechanical properties obtained from the stress-strain curves of PU films.

Polyurethane-Urea Film	Yield Stress (MPa)	Yield Strain (%)	Tensile Strength (MPa)	Elongation At-Break (%)
PU	14 ± 1	7 ± 1	34 ± 4	725 ± 16
PU+0.04 wt % GO in polyol	23 ± 1	3 ± 1	20 ± 5	637 ± 40
PU+0.04 wt % GO after prepolymer	19 ± 5	11 ± 3	33 ± 5	728 ± 41
PU+0.04 wt % GO in water	13 ± 1	13 ± 1	54 ± 3	695 ± 3

**Table 10:** Water contact angle values at 25 °C on PU films.

Polyurethane-Urea Film	Water Contact Angle (°)
PU	40 ± 0
PU+0.04 wt % GO in polyol	45 ± 2
PU+0.04 wt % GO after prepolymer	50 ± 2
PU+0.04 wt % GO in water	54 ± 4

**Table 11:** T-peel strength values of plasticized PVC/PUD/plasticized PVC joints. Locus of failure: CS—Cohesive failure of the substrate surface; CA—Cohesive failure of the adhesive; S—Rupture of substrate.

Polyurethane-Urea Dispersion	T-peel Strength—1 h (kN/m)	T-peel Strength—72 h (kN/m)	Locus of Failure
PUD	4.6 ± 0.4	6.2 ± 0.7	CA+CS
PUD+0.04 wt % GO in polyol	6.9 ± 0.7	12.0 ± 2.0	S+CA
PUD+0.04 wt % GO after prepolymer	7.4 ± 0.1	9.3 ± 0.1	CS+CA
PUD+0.04 wt % GO in water	6.3 ± 0.4	7.1 ± 0.3	CS+CA

**Table 12:** Single lap-shear values of stainless steel/PUD/stainless steel joints obtained 5 days after joints formation. Locus of failure: A—Adhesion failure.

Polyurethane-Urea Dispersion	Shear Strength (kPa)	Locus of Failure
PUD	95 ± 10	A
PUD+0.04 wt % GO in polyol	226 ± 29	A
PUD+0.04 wt % GO after prepolymer	190 ± 60	A
PUD+0.04 wt % GO in water	721 ± 49	A

## References

[1] Pérez-Limiñana M.A., Arán-Aís F., Torró-Palau A.M., Orgilés-Barceló A.C., Martín-Martínez J.M. (2005). Characterization of waterborne polyurethane adhesives containing different amounts of ionic groups. Int. J. Adhes. Adhes..

[2] Fuensanta M., Khoshnood A., Llansola F.R., Martín-Martínez J.M. (2020). New waterborne polyurethane-urea synthesized with ether-carbonate copolymer and amino-alcohol chain extenders with tailored pressure-sensitive adhesion properties. Materials.

[3] Feng H., Wang X., Wu D. (2014). Fabrication of spirocyclic phosphazene epoxy-based nanocomposites with graphene via exfoliation of graphite platelets and thermal curing for enhancement of mechanical and conductive properties. Ind. Eng. Chem. Res..

[4] Zhang X., Alloul O., He Q., Zhu J., Joseph M., Li Y., Wei S., Guo Z. (2013). Strengthened magnetic epoxy nanocomposites with protruding nanoparticles on the graphene nanosheets. Polymer (Guildf.).

[5] Giuri A., Colella S., Listorti A., Rizzo A., Mele C., Esposito Corcione C. (2018). GO/glucose/PEDOT:PSS ternary nanocomposites for flexible supercapacitors. Compos. Part B Eng..

[6] Sun M., Wang G., Yang C., Jiang H., Li C. (2015). A graphene/carbon nanotube π-conjugated polymer nanocomposite for high-performance organic supercapacitor electrodes. J. Mater. Chem. A.

[7] Ghaleb Z.A., Mariatti M., Ariff Z.M. (2014). Properties of graphene nano powder and multi-walled carbon nanotube-filled epoxy thin-film nanocomposites for electronic applications: The effect of sonication time and filler loading. Compos. A Appl. Sci. Manuf..

[8] Sharmila T.K.B., Nair A.B., Abraham B.T., Beegum P.M.S., Thomas E. (2014). Microwave exfoliated reduced graphene oxide epoxy nanocomposites for high performance applications. Polymer (Guildf.).

[9] Musico Y.L.F., Santos C.M., Dalida M.L.P., Rodrigues D.F. (2013). Improved removal of lead (II) from water using a polymer-based graphene oxide nanocomposite. J. Mater. Chem. A.

[10] Veeramalai C.P., Li F., Xu H., Kim T.W., Guo T. (2015). One pot hydrothermal synthesis of graphene like MoS_2_ nanosheets for application in high performance lithium ion batteries. RSC Adv..

[11] Pokharel P., Truong Q., Lee D.S. (2014). Multi-step microwave reduction of graphite oxide and its use in the formation of electrically conductive graphene/epoxy composites. J. Compos. B Eng..

[12] Strankowski M., Korzeniewski P., Strankowska J., Anu A.S., Thomas S. (2018). Morphology, mechanical and thermal properties of thermoplastic polyurethane containing reduced graphene oxide and graphene nanoplatelets. ACS Mater..

[13] Strankowski M. (2018). Effect of variation of hard segment content and graphene-based nano filler concentration on morphological, thermal, and mechanical properties of polyurethane nanocomposites. Int. J. Polym. Sci..

[14] Urban M., Strankowski M. (2017). Shape memory polyurethane materials containing ferromagnetic iron oxide and graphene nanoplatelets. Materials.

[15] Menes O., Cano M., Benedito A., Giménez E., Castell P., Maser W.K., Benito A.M. (2012). The effect of ultra-thin graphite on the morphology and physical properties of thermoplastic polyurethane elastomer composites. Compos. Sci. Technol..

[16] Gaidukovs S., Kampars V., Bitenieks J., Bochkov I., Gaidukova G., Cabulis U., Kampars V., Bitenieks J., Bochkov I., Gaidukova G. (2016). Thermo-mechanical properties of polyurethane modified with graphite oxide and carbon nanotube particles. J. Renew. Mat..

[17] Pokharel P., Lee D.S. (2014). High performance polyurethane nanocomposite films prepared from a masterbatch of graphene oxide in polyether polyol. Chem. Eng. J..

[18] Zhang F., Liu W., Liang L., Wang S., Shi H., Xie Y., Yang M., Pi K. (2020). The effect of functional graphene oxide nanoparticles on corrosion resistance of waterborne polyurethane. Colloids Surf. A Physicochem. Eng. Asp..

[19] Cai D., Jin J., Yusoh K., Rafiq R., Song M. (2012). High performance polyurethane/functionalized graphene nanocomposites with improved mechanical and thermal properties. Compos. Sci. Technol..

[20] Ding J.N., Fan Y., Zhao C.X., Liu Y.B., Yu C.T., Yuan N.Y. (2011). Electrical conductivity of waterborne polyurethane/graphene composites prepared by solution mixing. J. Compos. Mater..

[21] Lee Y.R., Raghu A.V., Jeong H.M., Kim B.K. (2009). Properties of waterborne polyurethane/functionalized graphene sheet nanocomposites prepared by an in situ method. Macromol. Chem. Phys..

[22] Kim H., Lee S. (2017). Electrical properties of graphene/waterborne polyurethane composite films. Fibers Polym..

[23] Zhang F., Liu W., Wang S., Jiang C., Xie Y., Yang M., Shi M. (2019). A novel and feasible approach for polymer amine modified graphene oxide to improve water resistance, thermal, and mechanical ability of waterborne polyurethane. Appl. Surf. Sci..

[24] Wan T., Chen D. (2019). In situ reduction of graphene oxide in waterborne polyurethane matrix and the healing behavior of nanocomposites by multiple ways. J. Polym. Sci. Part B Polym. Phys..

[25] Kim H., Miura Y., Macosko C.W. (2010). Graphene/polyurethane nanocomposites for improved gas barrier and electrical conductivity. Chem. Mater..

[26] Singh V., Joung D., Zhai L., Das S., Khondaker S.I., Seal S. (2011). Graphene based materials: Past, present and future. Prog. Mater. Sci..

[27] Wang X., Hu Y., Song L., Yang H., Xing W., Lu H. (2011). In situ polymerization of graphene nanosheets and polyurethane with enhanced mechanical and thermal properties. J. Mater. Chem..

[28] Fu Y.X., He Z.X., Mo D.C., Lu S.S. (2014). Thermal conductivity enhancement of epoxy adhesive using graphene sheets as additives. Int. J. Therm. Sci..

[29] Marami G., Nazari S.A., Faghidian S.A., Vakili-Tahami F., Etemadi S. (2016). Improving the mechanical behavior of the adhesively bonded joints using RGO additive. Int. J. Adhes. Adhes..

[30] Choi J.Y., Kim S.W., Cho K.Y. (2014). Improved thermal conductivity of graphene encapsulated poly(methyl methacrylate) nanocomposite adhesives with low loading amount of graphene. Compos. Sci. Technol..

[31] Zhao Z., Guo L., Feng L., Lu H., Xu Y., Wang J., Xiang B., Zou X. (2019). Polydopamine functionalized graphene oxide nanocomposites reinforced the corrosion protection and adhesion properties of waterborne polyurethane coatings. Eur. Polym. J..

[32] Nine M.J., Tran D.N.H., El Mekawy A., Losic D. (2017). Interlayer growth of borates for highly adhesive graphene coatings with enhanced abrasion resistance, fire-retardant and antibacterial ability. Carbon.

[33] Yang F., Wu Y., Zhang S., Zhang H., Zhao S., Zhang J., Fei B. (2020). Mechanical and thermal properties of waterborne polyurethane coating modified through one-step cellulose nanocrystals/graphene materials sols method. Coatings.

[34] Kale M.B., Luo Z., Zhang X., Dhamodharan D., Divakaran N., Mubarak S., Wu L., Xu Y. (2019). Waterborne polyurethane/graphene oxide-silica nanocomposites with improved mechanical and thermal properties for leather coatings using screen printing. Polymer.

[35] Cristofolini L., Guidetti G., Morellato K., Gibertini M., Calvaresi M., Zerbetto F., Montalti M., Falini G. (2018). Graphene materials strengthen aqueous polyurethane adhesives. ACS Omega.

[36] Tounici A., Martín-Martínez J.M. (2020). Addition of small amounts of graphene oxide in the polyol for synthesizing waterborne polyurethane-urea adhesives with improved adhesion properties. Int. J. Adhes. Adhes..

[37] Johra F.T., Lee J.W., Jung W.G. (2014). Facile and safe graphene preparation on solution based platform. J. Ind. Eng. Chem..

[38] Stankovich S., Dikin D.A., Dommett G.H.B., Kohlhaas K.M., Zimney E.J. (2006). Graphene-based composite materials. Nature.

[39] Giuri A., Masi S., Colella S., Listorti A., Rizzo A., Kovtun A., Dell’Elce S., Liscio A., Esposito Corcione C. (2017). Rheological and physical characterization of PEDOT:PSS/graphene oxide nanocomposites for perovskite solar cells. Polym. Eng. Sci..

[40] Strankowski M.B., Damian W.B., Piszczyk A., Strankowska J. (2016). Polyurethane nanocomposite containing reduced Graphene Oxide. FTIR, Raman and XRD Studies. J. Spectrosc..

[41] Potts J.R., Dreyer D.R., Bielawski C.W., Ruoff R.S. (2011). Graphene-based polymer nanocomposites. Polymer (Guildf.).

[42] Wan T., Chen D. (2018). Mechanical enhancement of self-healing waterborne polyurethane by graphene oxide. Prog. Org. Coat..

